# Supplementary material for: Examining the Role of the Noradrenergic Locus Coeruleus for Predicting Attention and Brain Maintenance in Healthy Old Age and Disease: An MRI Structural Study for the Alzheimer’s Disease Neuroimaging Initiative
Source: Cells. 2021 Jul 20;10(7):1829. doi: 10.3390/cells10071829 (PMC8306442; doi:10.3390/cells10071829)
Supplement: Supplementary file 1 [file cells-10-01829-s001.zip › cells-1207922-supplementary.pdf]

Supplementary Materials

Extended methods section

Data used in the preparation of this article were obtained from the Alzheimer's Disease Neuroimaging Initiative (ADNI) database (adni.loni.usc.edu). The ADNI was launched in 2003 as a public-private partnership, led by Principal Investigator Michael W. Weiner, MD. The primary goal of ADNI has been to test whether serial magnetic resonance imaging (MRI), positron emission tomography (PET), other biological markers, and clinical and neuropsychological assessment can be combined to measure the progression of mild cognitive impairment (MCI) and early Alzheimer's disease (AD).

<u>2.1. Dataset</u>

The data was downloaded from IDA (image data Archive powered by LONI - https://ida.loni.usc.edu/ - ADNI – Study Data) [49, 50]. The downloaded datasets of ADNI phases were merged for phase 2 and 3 in one Excel file outcoming from "NEUROBAT.csv", "PTDEMOOGGRAPHIC.csv" and "DXSUM_PDXCON_ADNIALL.csv". The "RID" (subjects' ID number) column was used to match the subjects with their MRIs. Analyses were carried out only on subjects at baseline ("VISCODE" – bl), hence, the MRIs that were selected were close in time (within six months approximately) to the date of the baseline administration of the Neuropsychological battery ("EXAMDATE"). The subjects were ordered for diagnosis and phase, then, subjects lacking Trail Making Test in the neuropsychological battery or other relevant sociodemographic information were removed.
In order to perform analyses investigating possible involvement of Job's complexity in Reserve and ageing, the subjects' job reported in the ADNI ("PTDEMOGRAPHIC.csv" – PTWORK and PTWRECNT) was converted into a continuous scale. The Job conversion was made according to the USA "Descriptions of skill levels from Standard Occupational Classification 2010" (https://www.bls.gov/soc/2010/2010_major_groups.htm), so, the jobs were ranked on a likert scale of 4 points from "less to more challenging cognitive demand and job complexity" required by the position held (examples: 1 = postal workers, cleaners; 2 = machine operation, caring occupation; 3 = protective service occupations, business and public service associate professionals; 4 = research and technology professionals, teaching and educational professionals). The conversion was made by an American Psychologist master student in TCD, blinded to the research hypotheses. The previous (PTWORK) and the last job performed (PTWRECENT), once converted to an ordinal scale, were summed together in order to obtain an "overall" measure of lifetime Job complexity and cognitive demand for each subject across the three groups.

<u>2.2. Statistical Analyses in JASP</u>

Analyses in JASP (https://jasp-stats.org/) were performed to investigate how the three groups varied. The first aim in JASP was to check significant differences between the groups in attentional visuo-motor speed processing measured with Trail Making Test – A. It is well documented how all neuropsychological domains decline with age and how they are heavily impaired in dementigenous clinical populations. In JASP, an ANOVA model was selected where TMT-A was entered as

dependent variable and the condition (HC, MCI, AD) as fixed factor. Secondly, the same model was used to check for differences in Education across the groups. As previously mentioned, education seems to play a significant role in protecting from cognitive decline, and is a resilience factor that increases brain health across different psychological and neurological diseases. Therefore, higher educational level was hypothesized in healthy controls and progressive lower education in the MCI and AD. Thus, in a second model the Education was inserted as continuous dependent variable. Similarly, in a third ANOVA, the same model was used to explore whether there was a systematic decline in BrainPAD as it was expected across groups. Again, BrainPAD was entered as dependent variable. Additionally, a fourth ANCOVA was carried out in order to explore if groups were differing in ranks of Job complexity and cognitive demand, so the sum job ranks (previous and recent) were used as continuous variable. Lastly, two further ANOVAs were carried out on Age and Total Intracranial volume in order to explore group differences.

The last part of the statistical analysis in JASP was a Pearson Correlation Matrix across the three groups. The following continuous variables were entered including the condition (ranked with 1 = HC, 2 = MCI, 3 = AD): Age, BrainPAD, Education, Job cognitive demand, GM volume, WM volume, WM Hyperintensities index, LC volume, VTA volume, Nucleus Basalis of Meynert/Substantia Innominata volume, Median Raphe volume, Dorsal Raphe volume, Control Pontine ROI volume, TMT-A and condition. In a second instance but in the same way, Bayes factors (BF) were calculated for the six ROIs in relationship with TMT-A and BrainPAD. BF were generated in the JASP interface (Bayesian correlation model) on the base of the average signal intensity of the significant clusters outcoming from the VBM analyses (when the ROIs were surviving the statistical thresholds settled). The average signal intensity of the whole ROI was considered in the Bayesian correlation matrices only for the ROIs which showed any significant cluster of voxels in the VBM analyses.

2.3. NEUROIMAGING

The MRI data was provided by the Alzheimer's Disease Neuroimaging Initiative – ADNI (http://adni.loni.usc.edu). 3Tesla T1-weighted images in Nifti format of ADNI 2 and ADNI 3 phases were downloaded from IDA (image data Archive powered by LONI - https://ida.loni.usc.edu/). Baseline T1-images of all subjects were selected and organised according to diagnosis: Cognitive Normal (CN/HC), Mild Cognitive Impairment (MCI) and Alzheimer Disease (AD). Diagnostic criteria of the ADNI are described by Petersen et al. 2010 [80]. To detect major motion or reconstruction artefacts, the quality control of the images was carried out referring to the rating scale guidelines (1= poor, 2= fair, 3= good, 4= excellent) of the Human Connectome Project (HCP) (https://www.humanconnectome.org/). Subjects with low definition (excessive blurriness) and/or marked ringing, inhomogeneities and motion artefacts were removed from the dataset. Given the theoretical aim to investigate predominantly the pontine structures, particular attention was focused on the integrity of the brainstem. Therefore, scans with poor brainstem resolution or marked artefacts were removed even if the rest of the brain had excellent resolution. Conversely, scans with poor cortical and cerebellar resolution were kept when brainstem quality was good and not heavily encroached by artefacts. In order to normalise the anatomical images and run the segmentation process, the native space scans were manually reoriented in order to reduce the variability of different orientation caused by different acquisition scanners. The images were reoriented using the MatLab

toolbox SPM12 (https://www.fil.ion.ucl.ac.uk/spm/software/spm12). Namely, using the display function, the origin was settled corresponding to the Anterior Commissures ("Set Origin"), then reoriented ("Reorient") and finally the outcoming "reorient.mat" file was stored for each subject in the same folder of the T1 scans. Subsequently, the reoriented scans were segmented using CAT12 (Computational Anatomy Toolbox - http://www.neuro.uni-jena.de/cat/) implemented in SPM12. The segmentation was run following the default CAT12 settings, except for the voxel size that was settled at 1mm isotropic voxel size. Hence, initially scans were denoised ("SANLM denoising" – medium) and registered to the SPM12 tissue probability maps (TPMs) with the "Affine Regularisation" to the ICBM space template of European brains in the Montreal Neurological Institute (MNI) coordinate space (the non-Caucasian brains in the ADNI were not considered in this study). Then, global intensity correction was performed classifying the tissues in order to segment the Grey Matter (GM), the White Matter (WM) and the Cerebral Spinal Fluid (CSF) according to the TPMs. Subsequently, the scans were bias corrected, so the signal inhomogeneities and the noise were removed, and signal intensity was globally normalised. DARTEL regularisation and ROI estimation of 'neuromorphometrics' atlas were applied. Finally, the segmented images were modulated by scaling with the amount of volume changes caused by spatial registration to templates. Therefore, the total amount of tissue (GM and WM) remains the same as it was in the native scans. The CAT12 segmentation process was carried out separately for the three groups in three different segmentations with the same pipeline and parameters. After the segmentation, the subjects with a quality check segmentation score below 70% were discarded **(no more than 0.6% of the sample)** according to CAT12 reports of quality assurance rating scale (100-90 = A [Excellent]; 90-80 = B [Good]; 80-70 = C [Satisfactory]; 70-60 = D [Sufficient]; 60-50 = E [Critical]; 50-40 = F [unacceptable/failed]. Only 6.2% of the selected images were below 80% (B).

Brainstem pontine structures like the Locus Coeruleus (LC) appear to be represented mostly in the segmented and normalised WM. In fact, the Brainstem is predominantly structured by White Matter, however in it, neurons bodies are embedded as well. For that reason, the following processes and the analyses were performed not only using segmented and normalised WMs but the whole brain (processed GM + processed WM), in order to include also the neurons bodies of the pontine structures. The whole brain scans were used to check the sample homogeneity in CAT12, and the outliers were removed above two standard deviations. Then, the images were smoothed using SPM12 interface with a 2 mm^3 FWHM kernel. Lastly, in order to better account for individual volumetric variability in the Voxel Based Morphometry (VBM) analyses, the Total Intracranial Volume (TIV) was calculated for each subject using CAT12 interface (Statistical Analyses – Estimate TIV).

2.3.1. Region of interest (ROI) masks

All the ROIs were 1mm3 isotropic voxel size and oriented in the MNI space. The six ROIs were manually drawn voxel by voxel in the same matrix size (FOV) of 181x217x181 (the same of the preprocessed images outcoming from CAT12). This procedure was carried out in FSLeyes (orhto-view – edit mode) using atlases as references. Throughout the process the ROIs were symmetrically corrected from the original atlases in order to better account for the large individual variability across the ADNI dataset and to reduce possible biases of "induced lateralization". The binary mask of six

ROIs outcoming from this process had the following number of voxels: Locus Coeruleus (LC) 714, Median Raphe (MR) 108, Dorsal Raphe (DR) 174, Ventral Tegmental Area (VTA) 252, Nucleus Basalis of Meynert (NBM) 494, Control Pontine region (CP) 906. The technical proceedings behind the ROIs definition are described in the following section for each neurotransmitter seed.

2.3.2. Locus Coeruleus ROI definition and binary mask

The accurate localization of the LC in human brain MRI is currently matter of study [33]. In the last few years, several probabilistic maps of the LC have been released, however, these probabilistic maps are inconsistent in both localization and volume extent within the MNI space. Indeed, different sample sizes have been recruited and this exacerbates the limitations due to different methodologies involved. These differences reflect a large anatomical variability of the samples scanned, suggesting that the LC varies across the general population. In order to perform volumetric analyses appropriate to the present research, and to attempt to resolve these differences, it was necessary to define a common space that included all the previous maps as to increase the likelihood of inclusion of the entirety of the LC, given the probable increase in between-subject anatomical variability in the present ADNI populations (n=395 HC, n=156 MCI, n=135 AD).

Therefore, a new symmetrical "omni-comprehensive" LC mask in the MNI space was created in order to include the whole LC rostro-caudal extent. Indeed, it was observed that with increasing age the LC signal intensity shifts from rostral to caudal portion [33, 81]. This process might be influenced by manifold variables, as ageing, the degree of biological brain maintenance and even dementia progression, which is likely to exacerbate this "caudal-shifting" process. Moreover, it is acknowledged how the noradrenergic system is susceptible to compensatory changes across the brain involving the caudal portion of the LC and peri-coeruleus/LC-peri-dendritic regions (Epi-coeruleus and Sub-coeruleus) [106, 128, 177, 178, 180, 14, 181, 182]. Therefore, considering a larger area rather than a very specific and concise region would be more informative and appropriate while investigating the LC-NA system on different groups, particularly known the heterogeneity of Alzheimer's disease.

The new "omni-comprehensive" LC map in the beginning considered four of the six LC probabilistic MNI atlases: 1) Keren et al. 2009 [81]; 2) Tona et al. 2017 [82] 3) Betts et al. 2017 [83]; Rong Ye et al. 2020 [84]. These maps and their respective methodologies are described below:

The Keren's 2SD LC mask was downloaded by the Eckert Laboratory website (http://eckertlab.org/lc/) [81]. The Keren's 2SD LC mask (0.5mm3 isotropic voxel size) oriented in the MNI space was obtained using 3T high-res scans of 44 healthy subjects between 19 and 79 years old. The "3T probabilistic Locus Coeruleus Atlas" provided by Tona et al. 2017 [82] was downloaded from NeuroImaging Tools & Resources Collaboratory website (https://www.nitrc.org/). The probabilistic high-res (0.5mm3 isotropic voxels size) atlas in MNI space was obtained scanning 17 healthy subjects (age range 19-24) in two sessions (2.8 months). The Betts' LC map (0.5mm3 voxels size) available in the supplementary materials of "*In vivo MRI assessment of the human locus coeruleus along its rostrocaudal extent in young and older adults*" [83] was obtained scanning in total 82 healthy subjects (age range 22-80) in a 3T MRI

scanner. Lastly, the Rong Ye [84] 7T LC probabilistic atlas (0.5mm3 voxels size) was downloaded from NeuroImaging Tools & Resources Collaboratory website (https://www.nitrc.org/). The probabilistic atlas was obtained scanning in an ultra-high filed 7T MRI scanner 53 healthy subjects in an age range between 52 and 84 years old.

Comparing the four atlases, Tona and Keren's maps have larger extensions compared with Betts and Rong Ye. The spatial localization of Eckert Lab's LC mask is anatomically smaller and represented more ventro-caudally and laterally than the Tona's probabilistic map, which is localized moderately more dorso-rostrally. While the Betts map is more represented in the caudal-dorsal portion of the Pons, the Rong Ye map instead has a smaller rostro-caudal extension than the previous maps and it is more shifted towards the walls of the 4th ventricle**.** Rong Ye 7T LC atlas overlaps a region of MNI space similar to the regions defined by Dahl 2019 and Liu 2019. These two atlases defined regions of the MNI space significantly smaller than the previous ones. For this reason, they were not practically considered during the processes for the new mask. However, it should be considered that these two atlases possibly highlight the higher rate of probability to identify the core of the LC since they involved larger samples than the others. Indeed, Dahl LC mask [32] was obtained scanning 299 subject from the Berlin Aging Study II (BASE-II) (more info can be found in his work "*Rostral locus coeruleus integrity is associated with better memory performance in older adults")*, and Liu LC mask [183] was obtained from 605 subjects (age range 18-88) from the Cambridge Centre for Ageing and Neuroscience (Cam-CAN) cohort (more info can be found in her work "*In vivo visualization of age-related differences in the locus coeruleus*"). It is worth mentioning, these two atlases were well matching the meta-mask developed by Dahl et al. (2021) [57], which considered the common regions of the MNI space defined by the previous six LC atlases. However, this recent work excludes the caudal portions defined by Betts, Tona and Keren, and the latter is the only histologically validated [179, 33] LC MRI atlas. Moreover, all of the previous six atlases are asymmetric in their left and right portions of the rostro-caudal extent.

The new "omni-comprehensive" LC mask included the six LC MNI atlases previously published: 1) Keren et al. (2009) [81]; 2) Tona et al. (2017) [82] 3) Betts et al. (2017) [83]; 4) Liu et al. (2019) [183], 5] Dahl et al. (2019) [32], and 6) Rong Ye et al. (2020) [84] without encroaching the Median Raphe (MR) and the Dorsal Raphe (DR) defined by Beliveau et al. 2015 [85] and the cerebellar white matter. Additionally, the new created LC "omni-comprehensive" mask included also the LC meta-mask developed by Dahl et al. (2021) [57], but with a larger and symmetrical rostro-caudal extent as to avoid induced lateralization biases in the analyses. Indeed, as pointed out by Betts et al. [83], the LC asymmetries reported in certain MRI studies could be caused by MRI biases of how radiofrequencies are transmitted and received in the scanner. In fact, post-mortem histological studies consistently revealed symmetrical distribution of LC cells [184, 185, 186, 187, 189, 14].

In order to obtain a binary mask suitable for Voxel Based Morphometry analyses in SPM12 (Statistical Parametric Mapping - https://www.fil.ion.ucl.ac.uk/spm/) using CAT12 (Computational Anatomy Toolbox 12), a new LC map of 1mm3 isotropic voxel size was manually drawn in FSL (https://fsl.fmrib.ox.ac.uk). The LC 1mm3 mask was drawn in the FSL edit mode (FSLeyes – Ortho View – Edit mode) in a blank nifti space of the size of 181x217x181 (the same size of the preprocessed

images outcoming from SPM12 and CAT12). The larger surfaces of the Tona and Keren atlases were used as main reference while considering and correcting for the spatial extensions of the other atlases. The drawing process was carried out by adding or removing voxels so to include the different anatomical localizations without overcoming the Dorsal and the Medial Raphe Nuclei, as defined by Beliveau et al. [85], and the cerebellar white matter. On completion of this procedure, the new bilateral LC mask was symmetrical and composed of 714 1mm3 voxels in a matrix size of 181x217x181. The new LC mask was anatomically inclusive of all the six previous atlases plus the meta-mask developed by Dahl (2021) [32]. Furthermore, the new LC mask was not encroaching any other pontine nuclei and the cerebellar vermal white matter (see Figure S1).

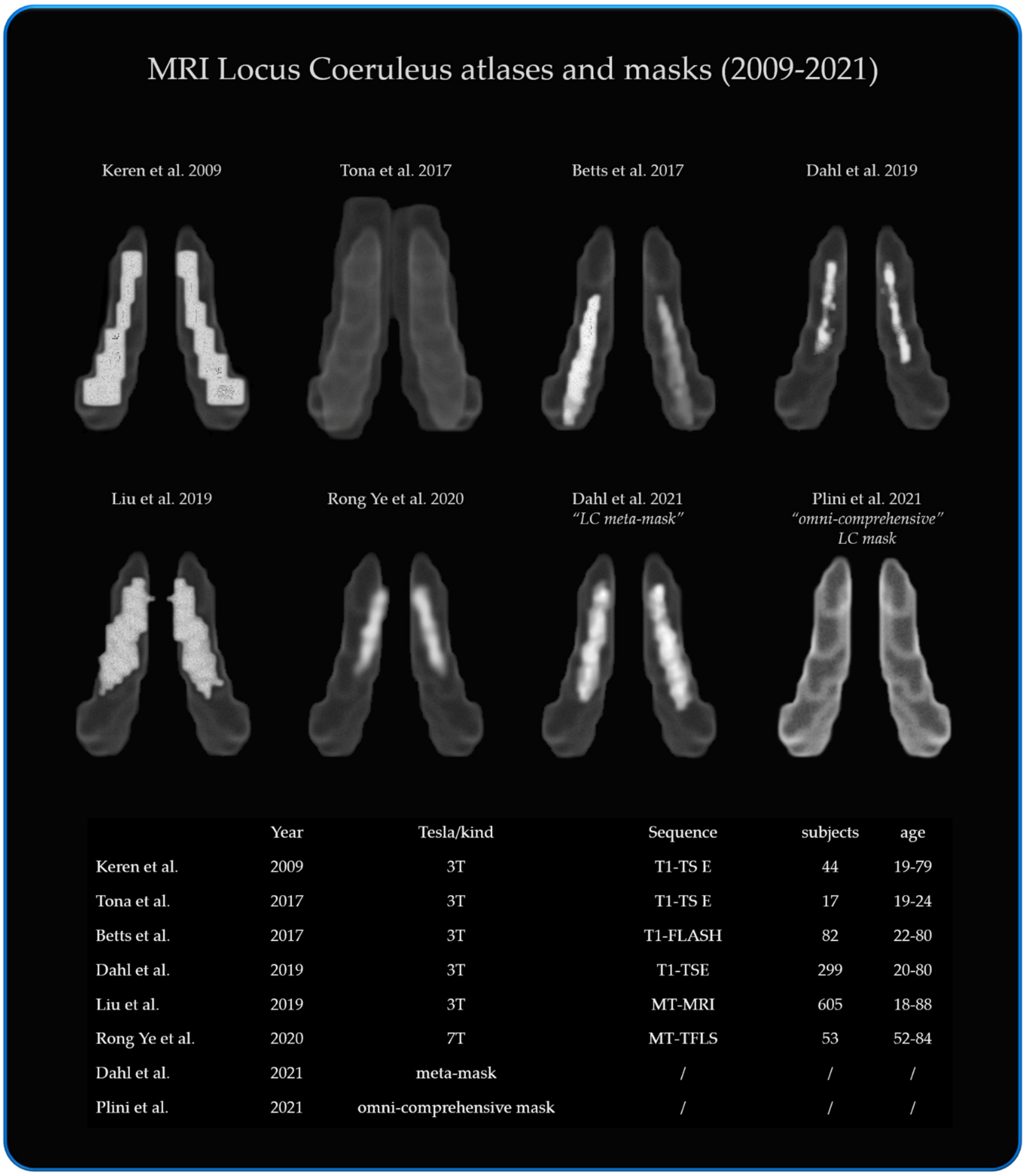

	Year	Tesla/kind	Sequence	subjects	age
Keren et al.	2009	3T	T1-TS E	44	19-79
Tona et al.	2017	3T	T1-TS E	17	19-24
Betts et al.	2017	3T	T1-FLASH	82	22-80
Dahl et al.	2019	3T	T1-TSE	299	20-80
Liu et al.	2019	3T	MT-MRI	605	18-88
Rong Ye et al.	2020	7T	MT-TFLS	53	52-84
Dahl et al.	2021	meta-mask	/	/	/
Plini et al.	2021	omni-comprehensive mask	/	/	/

Figure S1. Comparisons between the previously published Locus Coeruleus probabilistic atlases and the Locus Coeruleus mask created for the current study. The new LC mask was manually drawn merging the regions of MNI space defined by the atlases. The new mask was corrected in order to not encroach other pontine structures (Raphe Nuclei and cerebellar WM).

2.3.3. Raphe Nuclei ROIs definition and binary masks

Median and Dorsal Raphe Nuclei 1mm3 MNI masks were generously provided by Beliveau et al. (2017) "*A High-Resolution in vivo Atlas of the Human Brain's Serotonin System*" [85]. The probabilistic maps and masks were obtained by analysing 232 PET scans matched with high-res 3T structural MRI of healthy subjects between 18 and 45 years old. The provided Raphe Nuclei masks had a different size (193x229x193) from the CAT12 processed images (181x217x181 size). Therefore, in order to avoid a possible loss of accuracy in spatial resolution, the resampling to the 181x217x181 size using the flags "flirt" or "fnirt" in FSL was skipped. So, in FSLeyes using the "Edit mode" (FSLeyes – Ortho View – Edit mode), new 1mm3 symmetrical binary ROI exactly overlapping the Medial and Dorsal Raphe masks have been manually drawn, voxel by voxel, in another image originally displayed in a 181x217x181 size. Then, the resulting new MNI symmetrical Raphe masks had a size of 181x217x181 and 1mm3 isotropic voxel size. The new Median Raphe Nuclei 1mm3 MNI mask had a total of 108 voxels and the new Dorsal Raphe MNI mask a total of 174 voxels.

2.3.4. Ventral Tegmental Area ROI definition and binary mask

The Ventral Tegmental Area (VTA) mask was obtained by downloading the VTA MNI probabilistic map from the atlas made by Wolfgang M. Pauli et al. "*A high-resolution probabilistic in vivo atlas of human subcortical brain nuclei*" [86] from the NeuroVault website (https://neurovault.org/). The atlas was made using the MRI data from the Human Connectome Project (HCP) and was derived from a selected sample size of 168 healthy subjects between 22 and 35 years old. The image has a size of 193x229x193 different from the processed images from CAT12 (181x217x181 size). Hence, in order to avoid a possible loss of accuracy in spatial resolution, the resampling to the 181x217x181 size using the flags "*flirt*" or "*fnirt*" in FSL was skipped, as previously performed for the raphe nuclei. A new 1mm3 bilateral and symmetrical binary ROI exactly overlapping the bilateral VTA was manually drawn voxel by voxel in another image originally displayed in a 181x217x181 size in FSLeyes using the "Edit mode" (FSLeyes – Ortho View – Edit mode). Then, the resulting MNI bilateral 1mm3 VTA mask had 242 voxels in a size of 181x217x181.

2.3.5. Nucleus Basalis of Meynert (NBM) definition and binary mask

The localisation of the Substantia Innominata (SI) / Nucleus Basalis of Meynert (NBM) was more controversial than the previous nuclei as there are no specific maps available in MNI space. Albeit probabilistic MNI maps of the Acetylcholine cells of the Forebrain are provided by SPM Anatomy Toolbox 2.2c (https://www.fzjuelich.de/inm/inm1/EN/Forschung/_docs/SPMAnatomyToolbox/SPMAnatomyToolbox_node.html) [87], the probabilistic map referring to the SI/NBM (ch4.nii) is overlapping several subcortical nuclei delineated in other atlases. Therefore, the previous works of Kiliman et al. 2014 *Subregional Basal Forebrain Atrophy in Alzheimer's Disease: A Multicenter Study* [96], of George et al. 2011 *MRI-based volumetric measurement of the substantia innominata in amnestic MCI and mild AD* [68], Koulousakis et al. 2019 *The Nucleus Basalis of Meynert and Its Role in Deep Brain Stimulation for Cognitive*

Disorders: A Historical Perspective) [97] and Shultz et al. 2018 *Nucleus basalis of Meynert degeneration precedes and predicts cognitive impairment in Parkinson's disease* [69] were taken as comparative references in order to anatomically localise the SI/NBM and to better delineate it by integrating it with the probabilistic map of Acetylcholine in SPM Anatomy Toolbox defined by Zaborszky et al. *Stereotaxic probabilistic maps of the magnocellular cell groups in human basal forebrain* [87].

The Nucleus Basalis of Meynert was previously named also as Substantia Innominata of Reichert (SI), or Anterior Perforated Substance of Beccari, or even simply "magnocellular neurons within the basal Forebrain" [14, 95]. All of these names, however, are descriptive of the NBM as an intermediate medullary region in the basal Forebrain. The SI/NBM is placed ventrally to the Anterior Commissure and the Globus Pallidus of the Striatum and laterally to the rostral aspect of the Hypothalamus [14, 95]. As pointed out by Alan King Lun Liu et al. in "Nucleus basalis of Meynert revisited: anatomy, history and differential involvement in Alzheimer's and Parkinson's disease" [95], the Nucleus Basalis of Meynert lacks well-delineated boundaries and overlaps with other structures. Additionally, Acetylcholine cells are scattered around the forebrain from the Anterior Commissure to the Globus Pallidus.

Therefore, given this difficult and controversial localisation of the SI/NBM and the scattered Acetylcholine cells within the Forebrain, the Acetylcholine SPM Anatomy Toolbox 2.2c map (which was broadly including the whole probabilistic presence of Acetylcholine in the Forebrain) was used as main reference to delineate the SI/NBM, adjusting it by excluding the subcortical nuclei identified by other atlases and, at the same time, accounting for the probabilistic localisation of the SI/NBM delineated in previous studies. A new ROI was drawn voxel by voxel including the voxels of the SPM Acetylcholine map that were not overlapping other nuclei, and that were more spatially referring to the localisation and the anatomical shape of SI/NBM investigated in previous works. This procedure aimed to maximise the accuracy of the "control analyses on the neurotransmitter Acetylcholine", and to reduce possible "false positive" biases potentially outcoming for the inclusion of other important subcortical nuclei playing crucial roles not "strictly and directly related" to Acetylcholine synthesis and projection. In fact, the available Acetylcholine map in SPM Anatomy Toolbox was overlapping the internal and external portion of the Globus Pallidus, the Hypothalamus and Substantia Nigra Pars Compacta in the atlas of Pauli et al. *A high-resolution probabilistic in vivo atlas of human subcortical brain nuclei* [86]. Furthermore, the map was encroaching the Subthalamic Nuclei defined in the "Sub-Thalamic Nuclei atlas" available in FSLeyes [88], the Putamen and the Amygdala defined in the "MNI Structural Atlas" [89, 90] and the "Harvard-Oxford Cortical and Subcortical Structural Atlas" [91, 92, 93, 94] in FSLeyes. For this mask, a similar procedure to the previous mask was used. Using the "Edit mode" of FSLeyes (FSLeyes – Ortho View – Edit mode) on a previous image with a size of 181x217x181, a 1mm3 binary bilateral symmetrical mask was manually drawn by using the "Ch4" map from SPM Anatomy Toolbox as the main reference, excluding all the voxels that were overlapping the above-mentioned structures. The new mask was not overlapping any of the previous structures and seemed to match the previous anatomical rendering of SI/NBM. The SI/NBM MNI mask has a total amount of 494 1mm3 isotropic voxels.

2.3.6. Control Pontine ROI definition and binary mask

In order to control for a "neuromodulator-free" Brainstem's region, a squared binary ROI not referring to any anatomical nuclei was drawn in the ventro-rostral portion of the Pons. This additional control 1mm3 MNI ROI was manually drawn voxel by voxel in FLSeyes (FSLeyes – Ortho View – Edit mode) on the Template_T1_IXI555_MNI152_GS as the previous binary masks. The control pontine ROI has a size of 181x217x181 and a total amount of 906 voxels. The higher number of voxels was decided in order to obtain a control region closer to the number of voxels of the Locus Coeruleus and also capable of achieving a representative "control region" ideally more capable of detecting false positive results across the samples.

2.3.7. ROI's mean intensity extraction

In order to perform analyses in JASP (https://jasp-stats.org/) and obtain multiple correlation matrices, the average mean voxel intensity of each of the previous ROIs was extracted subject per subject across the three groups. The average values were extracted applying the 1mm3 binary masks of the six ROIs on the smoothed 2 mm^3 FWHM kernel whole brain images using FSL. In FSL the flags of "fslstats" "-k" (mask) and "-m" (output mean) were used to gather the average voxel intensities.

2.3.8. BrainPAD measure calculation

Brain Predicted Age Discrepancy (BrainPAD) is a measure of how the brain is ageing, and it is obtained by calculating the discrepancy between the chronological age and the biological age of the brain defined on healthy brain ageing trajectory of typical subjects. That means that subjects with a younger brain than their chronological age have negative values, whereas if a subject is ageing faster than its chronological age it has a positive value. BrainPAD is thought to reflect how well a brain is maintained, hence it is supposed to be an index of brain maintenance, i.e. a sub/complementary component of Reserve. In this case BrainPAD was calculated using only the Grey Matter, because it has been demonstrated that GM is more susceptible to brain ageing than WM, which is more stable in pathological conditions. BrainPAD measure developed by Boyle et al. [12] was developed using several datasets. In the first instance they defined the normal trajectory of GM ageing in healthy subjects. They then trained an algorithm to predict successfully the degree of GM deterioration in relation to the chronological age in further 3 populations of healthy subjects. The current study followed the whole process described in "*Brain-predicted age difference score is related to specific cognitive functions: a multi-site replication analysis*" by Rory Boyle et al. 2020 [12].

2.3.9. Voxel Based Morphometry (VBM) analyses

VBM analyses were performed using T1 whole brain images, after being pre-processed and smoothed 2 mm^3 FWHM kernel. Each set of analysis aimed to investigate five main research interests, based on Robertson's theoretical framework [1, 2]. Each analysis firstly considered the LC, and was then repeated separately for the other brainstem nuclei and the CP ROI as a control procedure testing both positive and negative relations in order to control for the opposite hypothesis. The statistical

thresholds were settled at *P<0.01*, and later increased progressively until the results disappeared (namely: *P<0.001*, *P<0.01 FWE*, *P<0.001 FWE*).

2.3.10. Relationship between LC volume and attention (VBM 1st branch)

The first question of this study was to investigate whether the LC volume can be associated with attentional performances measured with Trial Making Test A (visuo-motor speed processing). Three multiple regression models were run for each group. In these models, TMT-A was insert as continuous variable and TIV, Education and Age were insert as covariates. Then, based on previous literature and the main hypothesis, a negative relation between the LC volume and the TMT-A was also investigated, namely, larger LC volume was expected to be related to faster attentional performances (less seconds spent in completing the task). After the estimation of the statistical model ("estimating the statistical model") and after checking for the design orthogonality ("checking for design orthogonality"), the "results" were checked using the following contrast of SPM12: 0 0 0 0 -1. A positive relation with contrast: 0 0 0 0 1 was used as control analysis. A further step in the analysis was to indicate the LC mask as inclusive in order to isolate the LC involvement in the model. The same model was used for the three groups and applied to the other brainstem nuclei and the control pontine ROI separately as a control procedure to test both positive and negative relation.

2.3.11. Relationship between LC volume and brain maintenance (VBM 2nd branch)

In the second part of analyses, the previous pipeline was used to investigate the relation between LC volume and brain maintenance defined by BrainPAD construct. Once again, three separate multiple regression models were built in CAT12 for each group. In these models, TIV, Education and Age were considered as covariates, and BrainPAD was treated as dependent continuous variable. The negative relation between LC volume and BrainPAD was tested, hypothesizing that more LC volume is associated with lower and negative BrainPAD scores, which reflects a better ageing and a younger brain when compared to chronological age. Therefore, the contrast indicated in SPM12 was [0 0 0 0 -1]. The positive relation was considered as control analysis with the opposite contrast: [0 0 0 0 1]. Subsequently, in the VBM analyses pipeline the LC mask was applied as an inclusive mask in order to test the LC involvement in the model. Using the same models and contrasts for the three groups, the other Brainstem nuclei plus the control pontine ROI were investigated separately as control procedure for both positive and negative relations.

2.3.12. Relationship between LC volume and years of education (VBM 3rd branch)

The third group of analyses investigated the relation between years of Education and the volume of the LC. The multiple regression models were structured and carried out as described above. Education was entered as continuous dependent variable and age and TIV entered as continuous covariates. The positive relation between LC volume and education was tested as the main hypothesis, namely more years spent in education were related to greater LC volume. As control procedure, the opposite relation was investigated and the same was performed for the other ROIs.

2.3.13. Relationship between LC volume and occupational cognitive demand (VBM 4th branch)

The fourth branch of VBM analyses aimed to test the relation between the LC volume and years of education and the level of lifetime Job complexity and cognitive demand. It was explored whether higher ranks of Job complexity and cognitive demand were associated with higher LC volume. Following the same pipeline of the previous analyses, three different multiple regression models were built for the three groups. The positive relation between the LC volume and sum of the previous and the recent jobs performed by the subjects was investigated, accounting for TIV, Age and Education as covariates, and contrast [0 0 0 0 1]. As control analysis, the opposite was explored by using the contrast [0 0 0 0 -1]. Same model and parameters were applied for the other Brainstem nuclei and for the pontine control ROIs for both positive and negative relations.

2.3.14. Effect of cognitive decline on the ROIs volume (VBM 5th branch)

The analyses explored which of the 6 ROIs showed the greatest covariance with cognitive condition (HC, MCI, AD). In order to assess which ROIs presented the highest level of volume reduction, a full factorial statistical model was built in CAT12. The scans (whole brain WM+GM) of the three groups (HC, MCI, AD) were considered as the three levels of the factor "condition". The total intracranial volume, the age and the education were considered as continuous covariates. The six ROI masks were all merged in a same nifti file in order to be investigated in the model simultaneously. The merging procedure was carried out in FSL with the following command: "*fslmaths -add filename1.nii*". This command was applied to all the six ROIs, adding each ROI progressively.

As in the previous VBM analyses, the statistical model was built to investigate the volume difference between HC and MCI and between MCI and AD. Afterwards, the main effect of condition was explored across groups. The differences between groups and the main effect were investigated by considering the nifti file including the 6 ROI masks as inclusive mask.

2.3.15. Biological gender differences and neuromodulators' seeds volumes (VBM 6th branch)

Literature on gender differences concerning LC volume and its pathological courses is controversial [129, 31, 33, 168, 189]. Supplementary analyses have been carried out to assess volumetric differences due to the biological gender across the ADNI cohort. In CAT12 two-samples T-test models were carried out comparing males and females across the three groups (HC, MCI, AD). The VBM analyses were covaried for age, total intracranial volume and education. Again, the analyses were repeated without covariates in order to explore whether gender differences might be affected by the above-mentioned variables.

2.4. Mediation Analyses in SPSS using PROCESS

Mediation analyses with multiple parallel mediators were carried out in order to better clarify the possible role of the Locus Coeruleus as key component in mediating Reserve indices and attentional performances.
The analyses considered the three groups separately and were performed using the toolbox PROCESS v3.4 and SPSS macro developed by Andrew F.Hayes (http://www.processmacro.org/) implemented in SPSS 25 (https://www.ibm.com/products/spss-statistics). The toolbox PROCESS enables one to perform many different types of mediation and moderation analyses.

In SPSS, from the PROCESS interface it was selected the model number 4 with 95% confidence intervals and 5000 bootstrap samples. The model number enabled us to perform mediation analyses with parallel multiple mediators and covariates. The Trial Making Test A time in seconds was used as Y variable and BrainPAD scores as X variable. The extracted average volume values of the 6 ROIs were considered as six parallel mediators. the Total Intracranial Volume (TIV) and the age were treated as covariates. Similar to the statistical analyses made in JASP, the analyses were carried out separately on 395 Healthy Controls, 156 Mild Cognitive Impairments and 135 Alzheimer's Disease.

2.5 Rational for the neuromodulatory subcortical systems ROIs selection

From Dopamine, Noradrenaline is synthetized subcortically in the brainstem, specifically in the LC, in the dorsal pontine tegmentum and in the lateral tegmental neurons [14]. However, the LC is the most relevant neural basis of Noradrenaline, it is the main structure responsible for NA production and is in control of 90% of its cortical innervation. LC's projections are vastly spread throughout the cortex and the Cerebellum [15, 99]. For these reasons, it has been defined as the main core structure of investigation of the noradrenergic theory of cognitive reserve.

Given that the analyses were designed to be carried out also on clinical samples, control analyses were performed in order to account for the possibility to detect false positive concerning the LC involvement in attentional domain and Reserve due the ongoing diffuse neurodegeneration of the samples. Therefore, the other main neurotransmitters and their main core nuclei were considered as control regions as to better assess the implication the LC-NA system in Attention and Reserve.

The first control neurotransmitter defined for the control analyses was the serotoninergic system, because it is broadly supposed to modulate markedly different processes than Noradrenaline. Additionally, Serotonin is synthesised in the Pons very closely to the LC but from a different precursor (Tryptophan) [14], thus this anatomical area is particularly well suited for controlling purposes. The medial and the dorsal Raphe nuclei are the largest serotoninergic nuclei where serotonin is produced and then diffusely projected to the Cortex and the Cerebellum via the basal forebrain [14, 85].

Then, the Dopaminergic system was considered, and so the Ventral Tegmental Area was taken as a core area for the analyses. VTA is the main brain nucleus together with the Substantia Nigra (SN) where Dopamine is synthesised from the amino acid Tyrosine [14]. The VTA is responsible for the

cortical irroration of Dopamine while the SN is projecting subcortically. For this reason, VTA was defined as control region to control for the Dopaminergic system.

Regarding Acetylcholine, the Substantia Innominata – Nucleus Basalis of Meynert was chosen over the Tegmental Cholinergic Neurons, because it has the largest number of cholinergic neurons and it projects diffusely to whole brain cortex. The NBM is the largest cholinergic nuclei of the brain and over the 90% of the magnocellular neurons are cholinergic neurons [14, 95].

Finally, in order to control for a "neuromodulator-free" brainstem region, a ROI not referring to any anatomical nuclei was designed in the ventro-rostral portion of the Pons.

The analyses were designed to investigate the four main diffuse neurotransmitters systems: Noradrenaline (NA), Serotonin (5-HT), Dopamine (DA), Acetylcholine (ACh) and an additional neuromodulator-free control region.

Extended Results section

3.1. Sociodemographic characteristics

Age significantly differed across groups, $F(2,683) = 10.13$, $p < .001$. Post hoc comparisons showed that the HC group was significantly younger than the MCI group ($p = 0.014$) and the AD group ($p < 0.001$). The MCI and AD groups did not significantly differ from each other in age ($p = 0.68$).

Groups also differed in education, $F(2,683) = 19.41$, $p < .001$. The average years of education was greater for the HC group compared to the MCIs ($p < .001$) and the AD group ($p < .001$). The MCI and AD groups did not differ in mean years of education ($p = 0.68$). There was an effect of occupation, $F(2,683) = 11.71$, $p = 0.025$. The HC showed a higher occupational rank compared to MCI ($p=.031$). No other group comparisons for occupation were significant (all $p > 0.3$). Chi-squared test showed that gender was not evenly distributed across the three groups ($X^2 = 12.35$; df: 2; $p = 0.002$). As reported in the table 1, the HC contains significantly more females (57%) while in MCI and AD males are more highly represented (57.1% and 54.8% respectively).

3.2. Neural Indices

There was no significant difference in TIV by group, $F(2,683)=2.62$, $p=0.07$. However, there was a significant difference in BrainPAD scores, $F(2,683)=101.2$, $p < 0.001$. There was a systematic pattern in which AD patients showed greater BrainPAD scores (indicating an older brain relative to chronological age) compared to MCI patients ($p < 0.0001$), who in turn showed a greater mean BrainPAD score than the HC group ($p < 0.0001$). Average volume of the 6 subcortical ROIs were compared for HC, MCI and AD. ANCOVAs controlling for TIV demonstrated: **1**) a significant main effect of LC volume, $F(3,682)= 3.394$, $p=0.034$. Planned comparisons showed no significant difference

between HC and MCI (mean difference: 8.101e-4; T = 0.002; Pbonf= 1.000), no significant difference between AD and MCI (mean difference: -0.004; T = 0.002; Pbonf= 0.219), but a significant difference between AD and HC, (mean difference: -0.004, T = -2.598, Pbonf= 0.029). **2**) a significant main effect of DR volume, F(3,682)=19.35, p<0.001 across the three groups. Planned comparisons revealed that HC showed greater volume than MCI (mean difference: 0.005; T= 3.071; Pbonf= 0.007) and that MCI showed greater volume than the AD (mean difference: 0.006; T= 2.639; Pbonf: 0.025). The greatest difference was observed between HC and AD (mean difference: 0.011; T= 6.030; Pbonf <0.001). A similar trend was observed for the MR without reaching significance F(3,682)= 0.739, p= 0.478. **3**) a significant main effect for VTA, F(3,682)=9.969, p < 0.001. Planned comparisons showed no significant difference between HC and MCI in VTA volume (mean difference: 0.002; T= 1.655; Pbonf= 0.295), but significant difference between HC and AD (mean difference: 0.005; T= 4.437; Pbonf <0.001). Additionally, a significant difference of volume between MCI and AD (mean difference: 0.003; T= 2.427; Pbonf= 0.046) was observed. **4**) a main effect of the control pontine ROI F(3,682)=5.658, p=0.004. Planned two-way comparisons revealed a similar trend to the previous nuclei. HC showed greater volume than MCI (mean difference: 0.005; T= 2.525; Pbonf= 0.035), and also greater volume than AD (mean difference: 0.006; T= 2.813; Pbonf= 0.015). AD and MCI showed no significant volume difference (mean difference: -9.012e-4; T= 0.355; Pbonf= 1.000). **5**) no significant differences for MR, F(3,682)= 0.739, p= 0.478. HC – MCI (Mean difference: 9.201e-4; T= 0.535; Pbonf= 1.000), HC – AD (Mean difference: 0.002; T= 1.194; Pbonf: 0.699), MCI - AD (mean difference: 0.001; T= 0.581; Pbonf= 1.000). And **6**) no significant difference for NBM, F(3,682)=0.772, p=0.463. HC – MCI (mean difference: -7.3174e-4; T= 0.885; Pbonf= 1.000), HC – AD (mean difference: -9.379e-4; T= -1.075; Pbonf: 0.848), MCI - AD (mean difference: -2.061e-4; T= -0.200; Pbonf= 1.000). The average volumetric differences across the three groups for the six ROIs are reported in table S5.

3.3. Neuropsychological performance

All groups significantly differed in time taken to complete the TMT- A, F (2,683) = 127, p < 0.001. AD patients exhibited the longest duration (mean seconds) compared to the MCI (p < 0.001), which showed a longer mean time-to-completion than the HC group (p < 0.001). See table 1.

3.4. Biological gender differences and neuromodulators' seeds volumes

Analyses on gender found no significant differences across the three groups (see descriptive statistics table S6). More precise analyses as VBM analyses, see table S7 and section 3.11.

VBM analyses:

3.5. 1st branch VBM analyses: Multiple Regressions – TMT-A (attention – visuo-motor speed processing)

Does the LC predict attention performance relative to other neuromodulator seed regions?

As can be observed in table 2, in the HC group, 2 clusters of 15 voxels in the Nucleus Basalis of Meynert (NBM), 3 voxels of the Dorsal Raphe and 2 voxels of the Ventral Tegmental Area (VTA)

showed a significant negative relationship with TMT-A mean time-in-seconds at the p<0.01 threshold. By increasing the statistical threshold to p<0.001 only the 3 voxels of NBM survived. No voxels survived after FWE correction.

For the MCI group, reduced volume of the Locus Coeruleus (LC) was associated with longer TMT-A completion time. Specifically, 142 voxels of the LC were negatively related with TMT-A duration. Additionally, 9 voxels of the Dorsal Raphe and 1 voxel of the NBM were negatively associated with TMT-A duration. When the statistical threshold was raised to p<0.001 only 21 voxels of the LC survived. Nothing survived FWE correction.

Similarly, in the AD group, the strongest predictor of attention performance was the LC with 122 voxels negatively associated with the attentional performance. Further neurotransmitter seeds also contributed to the variance in TMT-A performance with 53 VTA voxels, 48 NBM voxels and 3 Dorsal Raphe voxels negatively associated with performance. At the statistical threshold of p<0.001, the LC, VTA and NBM were reduced to clusters of 9, 14 and 19 voxels respectively. All 3 neurotransmitter seeds did not survive when FWE correction was applied. As shown in the figure 3 the average LC results are localised within a region overlapping the LC core defined by previous atlases.

As an additional control procedure, the same analyses were repeated testing the opposite relationships (positive associations with TMT-A) across the 3 groups for all the 6 ROIs. These analyses show no significant results.

3.6. 2nd branch VBM analyses: Multiple Regressions - BrainPAD (Reserve – brain maintenance)
Does the LC predict brain maintenance relative to the other neuromodulator seed regions?

Table 3 shows a significant (p<0.01 threshold) cluster of 202 LC voxels predicting BrainPAD score in the HC group, demonstrating that greater LC volume is associated with a lower or negative (i.e. younger) BrainPAD score. Similarly, 89 voxels of the Dorsal Raphe nuclei, 5 voxels of the NBM and 2 voxels of the VTA predicted BrainPAD score. At the higher p<0.001 threshold, 153 LC voxels and 71 DR voxels predicted BrainPAD score with FWE correction. The VTA and NBM clusters did not survive at p<0.001 threshold.

The MCI group showed a similar trend to the HC group (without FWE correction). At the statistical threshold of p<0.01, 180 LC voxels and 130 DR voxels predicted BrainPAD score. A lesser contribution of 11 VTA voxels and 40 NBM voxels also predicted BrainPAD score. At the statistical threshold to p<0.001, all the 4 neurotransmitter seeds were reduced in their cluster extension (voxel count: LC=59; DR=109; VTA=2; NBM=12)

In the AD group the most widespread effects were observed. All the ROIs, except the control pontine region, were found to negatively predict to BrainPAD scores (without FWE correction). At the p<0.01 threshold, the most significant cluster was found in the LC (431 voxels). Two clusters of 145 and 52 voxels were observed in the Dorsal and Medial Raphe respectively. There were also 2 bilateral clusters in the VTA (33 voxels) and 3 bilateral clusters in the NBM (58 voxels). Increasing the statistical threshold to p<0.001 reduced the number of predictive voxels: LC=192; DR=90; MR=20 VTA=9;

NBM=5. As shown in figure 4, the average LC results are localised within a region overlapping the LC core defined by previous atlases.

As additional control procedure, the same analyses were repeated testing the opposite relation (positive relationship with BrainPAD) across the 3 groups for all the 6 ROIs. These analyses showed no significant results.

3.7. Mediation analyses

Does the LC mediate the relationship between BrainPAD (X) and attention performance (Y)?

A multiple parallel mediation analysis was conducted for each of the three groups. Bootstrap confidence intervals were used to examine the role of the six subcortical nuclei in mediating the relationship between BrainPAD score and attention performance, while controlling for age and TIV (see figure 5 - schematic mediation pathways).

The models were significant in the three groups. In HC and AD the total effect of X on Y was significant. The direct effects of X on Y were found not significant for the HC and significant for AD groups indicating that better brain maintenance relative to chronological age was predictive of attention performance (HC total effect of X on Y: 0.1586; se: 0.0552; T:2.874, p= 0.0043; LLCI: 0.0501; ULCI: 0.2672; c_ps: 0.0179; c_cs: 0.1378) (HC direct effect of X on Y: 0.1225; se: 0.0877; T:1.3979, p= 1.630; LLCI: -0.0498; ULCI: 0.2949; c_ps: 0.0138; c_cs: 0.1065). (AD total effect of X on Y: 1.7088; se: 0.3918; T:4.3608, p< 0.0000; LLCI: 0.9336; ULCI: 2.4840; c_ps: 0.0467; c_cs: 0.4262) (AD direct effect of X on Y: 1.2410; se: 0.4658; T:2.6644, p= 0.0088; LLCI: 0.3189; ULCI: 2.1630; c_ps: 0.0339; c_cs: 0.3095). However, no indirect effects of the 6 mediators were apparent.
However, in MCI the LC alone was found to significantly mediate the relationship between BrainPAD (Y) and TMT-A (X) (indirect effect of X on Y: 0.0927; BootSE: 0.0499; BootLLCI: 0.0111; BootULCI: 0.2043). The total effect of X on Y was also significant (effect: 0.4224; se: 0.1583; t: 2.6678; p: 0.0085; LLCI: 0.1096; ULCI: 0.7352; c_ps: 0.0237; c_cs: 0.2130). Controlling for the mediation effect, the direct effect of X on Y is not significant (direct effect: 0.0383; se: 0.2496; t: 0.1535; p: 0.8782, LLCI: -0.4552; ULCI: 0.5318, c'_ps: 0.0021, c'_cs:0.0193), implying that the effect of BrainPAD on attention performance in MCI is mediated indirectly through the LC volume. This finding suggests that the way that brain maintenance affects attention performance in MCI patients is disproportionately influenced by the noradrenergic system compared to other neuromodulatory systems.

3.8. Bayes Factors: parameters of evidence strength for the six ROIs involvement in attention and brain maintenance across the three groups

Bayes factors (BF) were calculated in order to better discriminate the differential involvement of the six ROIs in the two main domains investigated. BF_{10} confirmed the disproportional predictive involvement of the LC-NA system observed in the VBM analyses. Bayesian modelling demonstrated that LC volume exhibited the strongest relationship with BrainPAD and TMT-A (with the one exception of the LC - TMT-A correlation in the HC group). Overall, across the three groups, as

indicated by BF_{10}, the LC likelihood to predict brain maintenance is 19321,07 times higher than the null hypothesis, whereas it is 779,29 for the other five ROIs when summed together. Similarly, the LC likelihood to predict attention is 4158,30 times higher than the null hypothesis, while it is 241,35 for the sum of the other 5 ROIs. However, there are notable differences between the groups. Concerning BrainPAD, the MCI group compared to HC and AD shows the strongest evidence for LC (BF_{10} 19303,214), followed by DR (BF_{10} 399,634) and NBM (BF_{10} 339,646). Furthermore, it is noteworthy that there is evidence of absent relationships (support for the null hypothesis) for all other ROIs in the MCI group (BF_{10} < 1 indicating more evidence for the null hypothesis (i.e., no relationship). Similarly, for TMT-A, the LC shows the strongest evidence (BF_{10} 517,357), followed by the DR (BF_{10} 4,219), and for all the other ROIs there was no relationship. In the AD group the evidence for the LC is also the most substantial (BF_{10} 46,538) but there is also evidence for more distributed involvement of other nuclei as the DR (BF_{10} 7,031), the MR (BF_{10} 2,541) and the VTA (BF_{10} 4,657) supporting BrainPAD. In the same vein, for TMT-A the strongest evidence is in support of the LC (BF_{10} 3640,710), but strong evidence is also found for the VTA (BF_{10} 124,076) and the NBM (BF_{10} 102,490) and no evidence for the other ROIs. In the HC group the magnitude of the results was less pronounced but followed the same pattern. The LC shows the strongest evidence supporting BrainPAD (BF_{10} 142,324) followed by the DR (BF_{10} 22,466) and no evidence was found for all the other ROIs. Differently as how it was observed in the other groups, no evidence for the ROIs and TMT-A was found in the HC, with the only exception of the DR (BF_{10} 9,117). BF_{10} are reported specifically for the three groups in table 4 along with Pearson's correlation coefficients. BF_{10} are covaried for age, education and total intracranial volume.

3.9. 3rd and 4th branches of VBM analyses on education and occupational ranks

Do education and occupational status relate to brain reserve or maintenance?

The ROIs show only a negligible association with education and occupation (see table S5 and S6 in supplementary materials). Across the three groups, education and occupational ranks were correlated with each other (HC r= 0.418; MCI r=0.580; AD r=0.596; p<0.001), but neither education or occupational ranks were related with TMT-A (all p > 0.3) or BrainPAD (all p > 0.3), with the exception of the AD where education was weakly associated with TMT-A (r= -0.180; p= 0.02) and BrainPAD (r= -0.145; p= 0.093). Furthermore, the average volumes of the six ROIs showed no relationship with education and occupation across the 3 groups (all p > 0.08), except in the HC group where the DR and the control pontine region were found related to education (DR r= -0.131; p= 0.009; Control Pontine ROI r= -0.114; p= 0.024). However, in HC and MCI groups education was related with the overall brain volume (TIV) (HC r= 0.171; p<0.001; MCI r= 0.268; p<0.001) but this was not the case for AD (r= 0.142, p= 0.1).

3.10. 5th branch of VBM Analyses investigating structural differences between groups

Is there differential decline of the six subcortical nuclei across HC, MCI and AD groups?

3.10.1 Structural differences between HC and MCI patients

The significant ROIs where MCI show greater volume reduction than HC are reported in table 8. For the statistical threshold of p<0.001, the most significant volume difference is in clusters of 14 voxels distributed bilaterally in the VTA. The second most significant reduction is the control pontine ROI (18 voxels) and finally the DR (66 voxels). When FWE correction was applied none of the significant clusters survived.

3.10.2 Structural differences between MCI and AD patients

More distributed nuclei were reduced in volume in the AD group compared to the MCI group (see table 8). The most significant differences were found in 3 clusters, mostly right lateralized, in the LC involving a total number of 62 voxels. The second most significant difference was localised in the VTA in 2 left clusters of 12 voxels. A third cluster of 13 voxels was found in the DR. Lastly, only 1 voxel was found in the NBM. When the FWE correction was applied none of the significant clusters survived.

3.10.3. Main effect of factor condition (HC, MCI, AD) on the volume of the six ROIs

The main difference in terms of volume across clinical condition arises in cholinergic seed areas in two clusters of 50 voxels predominantly in the left portion of the NBM. The second region where the main effect of condition arises is in a cluster of 148 voxels in the DR. Additionally, 92 voxels in the VTA, 112 in the LC and 36 in the control pontine ROI are involved as well. The VBM findings are displayed in detail in table 7 for the statistical thresholds of p<0.001, in bold are highlighted the clusters surviving to p<0.05FWE (these findings did not survive when more conservative threshold was applied). See figure 6 for spatial resolution in the MNI space.

The above findings suggest a more limited deterioration of brainstem nuclei from HC to MCI than from MCI to AD. Moreover, there appears to be no evidence of volume reduction of the LC in MCI in keeping with a compensatory role of the LC as a mediator between brain maintenance, indexed by BrainPAD, and attention performance, measured by TMT-A. The observed volume reductions for these neuromodulators are consistent with the vast histopathological and neuroimaging literature concerning the Brainstem deterioration in neurodegenerative diseases [25, 26, 27, 30, 118, 31, 132, 133, 70, 68].

3.11. 6th branch of VBM analyses: Biological gender differences and neuromodulators' seeds volumes

Is there any difference due to gender for the six ROIs across HC, MCI and AD groups?

Analyses on gender found no significant differences across the three groups (see descriptive statistics table n.10). More precise analyses as VBM analyses (see table 11) showed negligible differences in the LC volumes for gender and more consistent differences for the VTA in HC group (females showed larger volume of the VTA compared to males). However, when the covariates TIV, age and education were removed from the models, the results became weaker or disappeared. This suggests that using the above-mentioned covariates in the five main branches of VBM analyses presented in the current

study is sufficient to account for the negligible gender differences uncovered by this 6th branch of VBM analyses. Overall, gender differences on neuromodulators' seeds were negligible across the three groups.

Table S1. Reports the VBM results predicting the Education score for each of the 6 ROI across the 3 groups (HC, MCI, AD). The results from the multiple regression models are covaried for age, education and total intracranial volume. The table shows the significant voxels for a statistical threshold of p<0.01.

**Brain Regions**	**side**	**MNI coordinates** *x*	*y*	*z*	peak **T** value [a]	peak **Z**-score [b]	peak cluster Ke [c]	p value uncorr [d]	FWE [e]	FDR [f]	total number of voxels for *p<0.01* threshold [g]
**HC** (n. 395)											
Locus Coeruleus	left	10	-40	-36	2.57	2.56	3	0.005	1.000	0.999	3
Dorsal Raphe	/	/	/	/	/	/	/	/	/	/	/
Median Raphe	/	/	/	/	/	/	/	/	/	/	/
Ventral Tegmental Area	/	/	/	/	/	/	/	/	/	/	/
Nucleus Basalis of Meynert	left	-16	-6	-10	2.50	2.49	2	0.006	1.000	0.999	2
Control Pontine ROI	/	/	/	/	/	/	/	/	/	/	/
**MCI** (n. 156)											
Locus Coeruleus	/	/	/	/	/	/	/	/	/	/	/
Dorsal Raphe	/	/	/	/	/	/	/	/	/	/	/
Median Raphe	/	/	/	/	/	/	/	/	/	/	/
Ventral Tegmental Area	/	/	/	/	/	/	/	/	/	/	/
Nucleus Basalis of Meynert	/	/	/	/	/	/	/	/	/	/	/
Control Pontine ROI	/	/	/	/	/	/	/	/	/	/	/
**AD** (n. 135)											
Locus Coeruleus	left	-8	-38	-34	2.79	2.75	4	0.003	1.000	0.999	5
Dorsal Raphe	/	/	/	/	/	/	/	/	/	/	/
Median Raphe	/	/	/	/	/	/	/	/	/	/	/
Ventral Tegmental Area	left	-2	-26	-22	2.52	2.48	2	0.007	1.000	0.999	2
Nucleus Basalis of Meynert	/	/	/	/	/	/	/	/	/	/	/
Control Pontine ROI	/	/	/	/	/	/	/	/	/	/	/

a) Peak T value: T value of the most significant cluster of contiguous voxels; b) Peak Z-score: Z-score of the most significant cluster of contiguous voxels; c) Peak cluster Ke: number of voxels of the most significant cluster of contiguous voxels; d) P value uncorrected; ;e) FWE = family wise error correction value; f) FDR = false discovery rate correction value ; g) Total number of voxels outcoming in the ROI including all clusters of contiguous voxels.

Table S2. Reports the VBM results predicting the Occupation score for each of the 6 ROI across the 3 groups (HC, MCI, AD). The results from the multiple regression models are covaried for age, education and total intracranial volume. The table shows the significant voxels for a statistical threshold of p<0.01.

**Brain Regions**	**side**	**MNI coordinates**			peak **T** value [a]	peak **Z**-score [b]	peak cluster Ke [c]	p value uncorr [d]	FWE [e]	FDR [f]	total number of voxels for *p<0.01* threshold [g]
		x	*y*	*z*							
**HC** (n. 395)											
Locus Coeruleus	/	/	/	/	/	/	/	/	/	/	/
Dorsal Raphe	/	/	/	/	/	/	/	/	/	/	/
Median Raphe	/	/	/	/	/	/	/	/	/	/	/
Ventral Tegmental Area	/	/	/	/	/	/	/	/	/	/	/
Nucleus Basalis of Meynert	/	/	/	/	/	/	/	/	/	/	/
Control Pontine ROI	/	/	/	/	/	/	/	/	/	/	/
**MCI** (n. 156)											
Locus Coeruleus	/	/	/	/	/	/	/	/	/	/	/
Dorsal Raphe	/	/	/	/	/	/	/	/	/	/	/
Median Raphe	/	/	/	/	/	/	/	/	/	/	/
Ventral Tegmental Area	right	6	-20	-18	2.80	2.56	2	0.005	1.000	0.997	7
Nucleus Basalis of Meynert	left	16	-2	-12	2.59	2.76	7	0.003	1.000	0.997	7
Control Pontine ROI	/	/	/	/	/	/	/	/	/	/	/
**AD** (n. 135)											
Locus Coeruleus	left	8	-40	-34	2.86	2.81	19	0.002	1.000	0.995	19
Dorsal Raphe	/	/	/	/	/	/	/	/	/	/	/
Median Raphe	/	/	/	/	/	/	/	/	/	/	/
Ventral Tegmental Area	/	/	/	/	/	/	/	/	/	/	/
Nucleus Basalis of Meynert	right	18	-2	-10	2.97	2.92	5	0.002	1.000	0.997	6
Control Pontine ROI	/	/	/	/	/	/	/	/	/	/	/

a) Peak T value: T value of the most significant cluster of contiguous voxels; b) Peak Z-score: Z-score of the most significant cluster of contiguous voxels; c) Peak cluster Ke: number of voxels of the most significant cluster of contiguous voxels; d) P value uncorrected; e) FWE = family wise error correction value; f) FDR = false discovery rate correction value; g) Total number of voxels outcoming in the ROI including all clusters of contiguous voxels.

Table S3. VBM full-factorial statistical model investigating the volume reduction of the 6 ROIs across the factor "condition" (HC, MCI, AD). In the table are shown the ROIs of reduced volume due the main effect of condition for a statistical threshold of p<0.001. The results are adjusted for total intracranial volume (TIV), age and education. In bold are highlighted the clusters survived when p<0.05 FWE was applied.

**Brain Regions**	**side**	**MNI coordinates**			peak **F** value [a]	peak **Z**-score [b]	peak cluster Ke [c]	p value uncorr [d]	FWE [e]
		x	*y*	*z*					

**Nucleus Basalis of Meynert**	**left**	**-16**	**-4**	**-10**	**33.89**	**7.66**	**35**	**0.000**	**0.000**
**Dorsal Raphe**	**right**	**2**	**-28**	**-6**	**28.66**	**7.02**	**132**	**0.000**	**0.000**
**Ventral Tegmental Area**	**left**	**-2**	**-18**	**-14**	**23.79**	**6.36**	**27**	**0.000**	**0.000**
**Locus Coeruleus**	**right**	**4**	**-36**	**-18**	**21.46**	**6.01**	**64**	**0.000**	**0.001**
**Ventral Tegmental Area**	**right**	**4**	**-22**	**-16**	**20.43**	**5.85**	**38**	**0.000**	**0.003**
Locus Coeruleus	left	-4	-36	-18	17.02	5.29	26	0.000	0.062
Nucleus Basalis of Meynert	right	18	-4	-10	16.12	5.13	3	0.000	0.129
Nucleus Basalis of Meynert	right	14	-6	-10	14.72	4.87	8	0.000	0.367
Nucleus Basalis of Meynert	right	10	-4	-10	10.89	4.08	2	0.000	1.000
Locus Coeruleus	right	8	-40	-30	10.19	3.92	22	0.000	1.000
Control Pontine	right	2	-24	-34	9.53	3.77	12	0.000	1.000
Control Pontine	right	6	-30	-34	8.45	3.49	8	0.000	1.000
Nucleus Basalis of Meynert	right	20	-2	-8	8.35	3.47	2	0.000	1.000
Control Pontine	right	4	-24	-24	7.46	3.23	2	0.001	1.000
Control Pontine	right	4	-28	-26	7.26	3.17	4	0.001	1.000

a) Peak T value: T value of the most significant cluster of contiguous voxels; b) Peak Z-score: Z-score of the most significant cluster of contiguous voxels; c) Peak cluster Ke: number of voxels of the most significant cluster of contiguous voxels; d) p value uncorrected; e) FWE = family wise error correction value; f) FDR = false discovery rate correction value (q).

Table S4. VBM full-factorial statistical model investigating the volume reduction of the six ROIs across the factor "condition" (HC, MCI, AD). The table shows the regions of significant reduced volume in MCI compared with HC for a statistical threshold of p<0.001. The lower portion shows the regions of significant reduced volume in AD compared with MCI. The results are adjusted for total intracranial volume (TIV), age and education.

**Brain Regions**	**side**	**MNI coordinates**			peak **T** value [a]	peak **Z**-score [b]	peak cluster Ke [c]	p value uncorr [d]	FWE [e]	FDR [f]
		x	*y*	*z*						
**HC>MCI**										
Ventral Tegmental Area	right	4	-22	-16	4.04	4.02	6	0.000	1.000	0.168
Control Pontine	right	2	-24	-34	3.78	3.76	18	0.000	1.000	0.298
Dorsal Raphe	left	-2	-28	-6	3.75	3.72	66	0.000	1.000	0.318
Ventral Tegmental Area	left	2	24	-24	3.48	3.46	8	0.000	1.000	0.537
**MCI>AD**										
Locus Coeruleus	right	4	-36	-18	4.75	4.71	30	0.000	0.537	0.014
Locus Coeruleus	left	-4	-36	-18	4.45	4.42	12	0.000	0.921	0.037
Locus Coeruleus	right	8	-40	-30	4.25	4.22	20	0.000	0.995	0.068
Ventral Tegmental Area	left	4	-18	-14	4.23	4.20	4	0.000	0.997	0.071
Ventral Tegmental Area	left	-2	-18	-14	4.19	4.16	8	0.000	0.999	0.082
Dorsal Raphe	right	4	-28	-6	3.84	3.82	5	0.000	1.000	0.212
Nucleus Basalis of Meynert	right	14	-6	-10	3.57	3.55	1	0.000	1.000	0.396
Dorsal Raphe	left	-2	-32	-8	3.53	3.51	6	0.000	1.000	0.435
Dorsal Raphe	right	2	-32	-8	3.45	3.43	2	0.000	1.000	0.514

a) Peak T value: T value of the most significant cluster of contiguous voxels; b) Peak Z-score: Z-score of the most significant cluster of contiguous voxels; c) Peak cluster Ke: number of voxels of the most significant cluster of contiguous voxels; d) p value uncorrected; e) FWE = family wise error correction value; f) FDR = false discovery rate correction value (q).

Table S5. Shows the average volumes of the six ROIs for the three groups (HC, MCI, AD). The average values were extracted from the whole brain images (GM+WM) smoothed 2 mm^3 FWHM kernel. More precise analyses are provided by the VBM analyses.

	Locus Coeruleus*		Dorsal Raphe Nucleus***		Median Raphe Nucleus		Ventral Tegmental Area***		Nucleus Basalis of Meynert		Control Pontine ROI**	
**Groups**	**mean**	**SD**	**mean**	**SD**	**mean**	SD	mean	SD	mean	SD	mean	SD
HC (395)	0,871	0,019	0,856	0,019	0,931	0,020	0,950	0,012	0,984	0,009	0,982	0,022
MCI (156)	0,871	0,014	0,851	0,017	0,931	0,016	0,948	0,012	0,985	0,008	0,977	0,022
AD (135)	0,867	0,014	0,846	0,016	0,929	0,015	0,945	0,011	0,985	0,010	0,976	0,018

(* $p < .05$; ** $p < .01$; *** $p < .001$)

Table S6. Shows the average volumes of the six ROIs for the three groups (HC, MCI, AD) divided by biological gender (1 males, 2 females). The average values were extracted from the whole brain images (GM+WM) smoothed 2 mm^3 FWHM kernel. More precise analyses are provided by the VBM analyses on bilogical gender.

Healthy Controls n=395

	Locus Coeruleus		Dorsal Raphe		Median Raphe		Ventral Tegmental Area		Nucleus Basalis of Meynert		Control Pontine ROI	
	**Male**	**Female**	Male	Female	Male	Female	Male	Female	Male	Female	Male	Female
**N**	**168**	**227**	168	227	168	227	168	227	168	227	168	227
**Mean**	0.8711	0.8717	0.8527	0.8588	0.9316	0.9314	0.9494	0.9503	0.9854	0.9838	0.9820	0.9814
**SD**	0.01388	0.02218	0.01786	0.01968	0.01466	0.02319	0.009798	0.01408	0.008573	0.008614	0.01414	0.02679
**Min.**	0.8200	0.6356	0.8039	0.6888	0.8891	0.6776	0.9149	0.7894	0.9644	0.9449	0.8989	0.6629
**Max.**	0.9013	0.9082	0.9013	0.8898	0.9644	0.9620	0.9673	0.9761	1.008	1.005	1.001	1.003

Mild Cognitive Impairment n=156

	Locus Coeruleus		Dorsal Raphe		Median Raphe		Ventral Tegmental Area		Nucleus Basalis of Meynert		Control Pontine ROI	
	Male	Female	Male	Female	Male	Female	Male	Female	Male	Female	Male	Female
**N**	89	67	89	67	89	67	89	67	89	67	89	67
**Mean**	0.8713	0.8700	0.8473	0.8550	0.9313	0.9301	0.9480	0.9482	0.9859	0.9846	0.9769	0.9760
**SD**	0.01353	0.01488	0.01662	0.01608	0.01554	0.01640	0.01201	0.01150	0.008076	0.008829	0.01925	0.02617
**Min.**	0.8305	0.8325	0.8131	0.8187	0.8916	0.8919	0.9088	0.9093	0.9650	0.9553	0.8774	0.8506
**Max.**	0.9053	0.9024	0.8905	0.8918	0.9676	0.9614	0.9723	0.9696	1.004	1.003	0.9995	1.000

Alzheimer's Disease n=135

	Locus Coeruleus		Dorsal Raphe		Median Raphe		Ventral Tegmental Area		Nucleus Basalis of Meynert		Control Pontine ROI	
	Male	Female	Male	Female	Male	Female	Male	Female	Male	Female	Male	Female
**N**	74	61	74	61	74	61	74	61	74	61	74	61
**Mean**	0.8670	0.8667	0.8410	0.8521	0.9279	0.9305	0.9426	0.9470	0.9859	0.9845	0.9753	0.9759
**SD**	0.01360	0.01383	0.01496	0.01633	0.01607	0.01373	0.009740	0.01113	0.009980	0.009445	0.01569	0.02024
**Min.**	0.8386	0.8262	0.8079	0.8090	0.8900	0.8807	0.9108	0.9039	0.9638	0.9547	0.9160	0.8960
**Max.**	0.8969	0.8911	0.8740	0.8843	0.9727	0.9526	0.9599	0.9671	1.038	1.009	0.9972	0.9979

Table S7. Results from VBM T-Test comparisons within the three groups (HC, MCI, AD). The table shows the number of significant voxels of difference between the two sub-groups (male vs female) across the three groups. The left column (M more than F) shows the number of voxels males have more than the females. The right column shows the number of voxels females have more than males.

The results are covaried for Age, Education and Total Intracranial Volume for 2 different statistical thresholds P<0.01 and P <0.001. All the results did not survive multiple comparison correction. When the same analyses were repeated without covariates the results became weaker or disappeared. This suggests that using the above-mentioned covariates is sufficient to account for gender differences.

	**Healthy Controls M (168) F (227)**				**Mild Cognitive Impairment M (89) F (67)**				**Alzheimer's Disease M (74) F (61)**			
	**M more than F**		F more than M		M more than F		F more than M		M more than F		F more than M	
	P<0.01	*P<0.001*	*P<0.01*	*P<0.001*	*P<0.01*	*P<0.001*	*P<0.01*	*P<0.001*	*P<0.01*	*P<0.001*	*P<0.01*	*P<0.001*
**LC**	6 T2.77	/	19 T2.56	/	1 T2.40	/	8 T3	/	1 T2.45	/	3 T2.67	/
**DR**	/	/	1 T2.51	/	/	/	33 T3.42	**5 T3.42**	/	/	125 T3.8	**20 T3.83**
**MR**	/	/	/	/	/	/	2 T2.96	/	/	/	/	/
**NBM**	3 T2.81	/	23 T3.52	**3 T3.52**	1 T2.42	/	/	/	/	/	8 T3.24	/
**VTA**	/	/	126 T4.73	**67 T4.66**	/	/	24 T3.69	**7 T3.69**	/	/	31 T3.19	**3 T3.19**
**CP**	38 T3.35	**2 T3.35**	/	/	13 T2.76	/	/	/	83 T3.58	**13 T3.58**	2 T2.55	/
